# Reducing disparities in behavioral health treatment in pediatric primary care: a randomized controlled trial comparing Partnering to Achieve School Success (PASS) to usual ADHD care for children ages 5 to 11 – study protocol

**DOI:** 10.1186/s12875-024-02473-7

**Published:** 2024-06-22

**Authors:** Jennifer A. Mautone, Alex Holdaway, Wendy Chan, Jeremy J. Michel, James P. Guevara, Amala Davis, Colette Desrochers, Erica Evans, Zia Gajary, Siobhan Leavy, Danah Rios, Katie L. Tremont, Jaclyn Cacia, Billie S. Schwartz, Abbas F. Jawad, Thomas J. Power

**Affiliations:** 1 Children’s Hospital of Philadelphia, Roberts Center for Pediatric Research, 2716 South Street, Philadelphia, PA 19146 USA; 2 Perelman School of Medicine at, University of Pennsylvania, Philadelphia, PA USA; 3 Graduate School of Education at University of Pennsylvania, Philadelphia, PA USA; 4 Chester County Intermediate Unit, Downingtown Philadelphia, PA USA; 5 Caregiver Partner, Philadelphia, PA USA

**Keywords:** (Three to ten) attention-deficit/hyperactivity disorder, Pediatric primary care, Behavior therapy, Family engagement, Reducing disparities

## Abstract

**Background:**

Integrating behavioral health services into pediatric primary care can improve access to care, especially for children marginalized by poverty and racial/ethnic minority status. In primary care, a common presenting concern is attention-deficit/hyperactivity disorder (ADHD). Services in primary care for marginalized children with ADHD typically include medication alone; therapy to improve skills and build relationships is less available. This study evaluates the effectiveness of a behavioral intervention offered through primary care for marginalized families coping with ADHD (Partnering to Achieve School Success, PASS) compared to treatment as usual (TAU).

**Method:**

Three hundred participants will be randomly assigned to PASS or TAU. Participants include children ages 5 to 11 who have ADHD and are from economically marginalized families. PASS is a personalized, enhanced behavioral intervention that includes evidence-based behavior therapy strategies and enhancements to promote family engagement, increase caregiver distress tolerance, and provide team-based care to improve academic and behavioral functioning. TAU includes services offered by primary care providers and referral for integrated behavioral health or community mental health services. Outcomes will be assessed at mid-treatment (8 weeks after baseline), post-treatment (16 weeks), and follow-up (32 weeks) using parent- and teacher-report measures of service use, child academic, behavioral, and social functioning, parenting practices, family empowerment, and team-based care. Mixed effects models will examine between-group differences at post-treatment and follow-up. Analyses will examine the mediating role of parenting practices, family empowerment, and team-based care. Subgroup analyses will examine differential effects of intervention by child clinical characteristics and socioeconomic factors.

**Discussion:**

This study is unique in targeting a population of children with ADHD marginalized by low socioeconomic resources and examining an intervention designed to address the challenges of families coping with chronic stress related to poverty.

**Trial registration:**

This study was registered on clinicaltrials.gov (NCT04082234) on September 5, 2019, prior to enrollment of the first participant. The current version of the protocol and IRB approval date is October 4, 2023. Results will be submitted to ClinicalTrials.gov no later than 30 days prior to the due date for the submission of the draft of the final research report to the Patient-Centered Outcomes Research Institute.

**Supplementary Information:**

The online version contains supplementary material available at 10.1186/s12875-024-02473-7.

## Background and rationale

Behavioral health conditions are estimated to occur among at least 20% of children and adolescents [1], but only about one in five children with significant mental health needs receive evidence-based services [2]. Disparities in service access and use are substantial; children marginalized by low family income who disproportionately belong to underrepresented racial/ethnic groups utilize mental health services significantly less than those who are more advantaged [3, 4].

Children from economically marginalized groups face numerous challenges that contribute to poorer health outcomes [5]. Poverty confers upon these children enormous risk, including chronic stress related to single parenting, frequent life transitions, parental psychopathology, and exposure to violence. These factors, in turn, are associated with disrupted parent–child attachments, negative parenting, and increased risk of comorbid child mental health conditions [6, 7]. Further, these children often attend schools that are under-resourced and stressed by staff shortages, high rates of staff turnover, and the challenge of supporting students living in poverty [8].

Lack of service use is a significant problem because untreated mental health conditions are associated with poor outcomes [9]. Pediatric primary care has become a major venue for the delivery of behavioral health services, in response to the urgent need to improve access to care and reduce disparities in service utilization. Behavioral health services in primary care target a broad range of conditions, including ADHD, given its high prevalence (estimated 9%) [10] and associated impairments [11]. In fact, up to 25% of referrals to integrated behavioral health in primary care providers are for concerns related to attention and school problems [12]. Children with ADHD are at risk for numerous adverse outcomes later in adolescence, including anti-social behavior and incarceration, school disengagement and drop out, [13–15] and more frequent medical visits [16]. Families of children with ADHD spend more money per year on health care for their children than families of children without the disorder [17]. In addition, relative to a typically developing child, the incremental cost to educate a child with behavior disorders is substantially higher largely due to costs associated with special education placement, grade retention, and disciplinary incidents [18]. In response to the enormous need of children with ADHD and ongoing problems with access to services, the American Academy of Pediatrics (AAP) has highlighted primary care as a major venue for assessment and management of this condition [19].

This study has a broad focus on the management of behavioral health conditions in primary care. ADHD is used as an exemplar [20] because of the feasibility of managing this disorder in primary care. This study addresses a high priority identified by the Institute of Medicine to compare the effectiveness of interventions based in primary care for children with ADHD [9].

The optimal strategy for improving outcomes for most children with ADHD includes behavior therapy in combination with medication [21–23]. Despite current medication shortages, medication may be easier to obtain through primary care than behavioral interventions, and the majority of children with ADHD (62%) receive medication [10]. In contrast, behavior therapy is less accessible; about 8% of families reported they currently receive this intervention and only 31% have ever received this service [10]. As a result, families of children with ADHD, particularly those who are economically marginalized, may have access to only one mode of treatment (medication), which may address symptoms but not critical areas of impairment and may not be acceptable or preferred by families [24].

Providing services in primary care can improve access [25] to behavioral health care. Although studies have generally demonstrated the effectiveness of behavioral interventions provided in primary care [26], research on the effectiveness of behavior therapy provided in primary care for marginalized children with ADHD is very limited. The studies conducted to date have not focused specifically on children with ADHD [27–29]; do not include enhancements tailored to economically marginalized families [27, 29]; and/or are too brief to include some critical components of evidence-based behavior therapy [28].

This study addresses a priority for comparative effectiveness research, listed in the first quartile by the Institute of Medicine: Examine the effectiveness of treatment strategies based in primary care for ADHD in children [9]. In this study, we will compare the effectiveness of enhanced behavior therapy offered in primary care, known as Partnering to Achieve School Success (PASS), to treatment as usual (TAU) informed by AAP guidelines for ADHD and facilitated by electronic practice supports as well as access to behavioral health services that are integrated into primary care practice. Enhancements to behavior therapy include strategies to integrate medical and behavioral health care, promote family motivation and engagement, support family-school-primary care collaboration, and support families in coping with chronic stress. Of note, PASS also includes the involvement of a Community Health Partner or patient navigator; research has shown that these individuals can be effective in improving patient engagement in behavioral health services [30]. The study is designed to examine the effectiveness of PASS in improving service use and outcomes important to families of children with this disorder, specifically academic performance, behavioral compliance, and interpersonal relationships.

### Study objectives

The study has four objectives. The first objective is to evaluate differences between PASS and TAU in improving utilization of ADHD services and patient-centered outcomes for children with ADHD from economically marginalized families at post-treatment (16 weeks after baseline) and follow-up (32 weeks after baseline). We hypothesized that, compared to TAU, families in PASS will show greater use of services for ADHD, and children in PASS will demonstrate greater improvements in academic performance, behavior compliance, interpersonal competence, life satisfaction, and ADHD symptoms.

Second, we will compare PASS and TAU on parenting practices, caregiver sense of empowerment to navigate systems of care, and caregiver perceptions of the quality of team-based care. It is hypothesized that, compared to TAU, families in PASS will show a greater reduction in negative parenting practices, an improvement in their sense of empowerment in being able to navigate care delivery systems, and an increase in their perceptions of team-based care. Third, we will explore whether changes in caregiver processes (parenting practices, caregiver sense of empowerment, perceptions of team-based care) mediate the effect of intervention on child outcomes. Finally, we will explore the heterogeneity of treatment effect for families of varying levels of socioeconomic resources, caregivers with varying levels of stress and health literacy, medicated versus unmedicated children, and children with varying levels of mental health symptom severity at baseline.

### Trial design

The study is a two-group, parallel randomized controlled trial**.** Patients will be randomly assigned to PASS or TAU, 150 participants per condition. Randomization will be stratified by practice (seven practices) and parental report of ADHD medication usage (yes, no) at time of random assignment. We will request that parents in PASS and TAU report about services they are receiving in the community during the intervention and follow-up periods. Outcome measures pertaining to Objectives 1 and 2 will be administered at baseline, post-treatment (16 weeks after baseline), and follow-up (32 weeks after baseline) from caregivers and teachers. Caregivers, but not teachers, will also be asked to complete a brief set of outcome measures at a mid-treatment time point (8 weeks after baseline). During the follow-up period, families in PASS will no longer receive the PASS intervention, and families in both conditions will have access to behavioral health services integrated into primary care. Family attendance at sessions and adherence to recommended treatment strategies will be assessed continuously during the intervention. Family utilization of behavioral health services in primary care prior to the study, during the intervention period, and during follow up will be monitored to examine the contrast in service delivery between treatment conditions. Subgroups identified by level of socioeconomic resources, caregiver stress, and caregiver health literacy, child clinical characteristics, as well as child medication status at baseline will be delineated to examine whether there are differences in outcome as a function of subgroup. PASS will be provided in the context of the integrated primary care program at our institution. Integrated primary care providers will incorporate PASS sessions into their clinical schedules, deliver PASS to families, and bill for services as they typically do in practice. To promote participant retention in the study, newsletters will be disseminated to caregiver participants on a regular basis.

### Conceptual framework

**Fig. 1:**
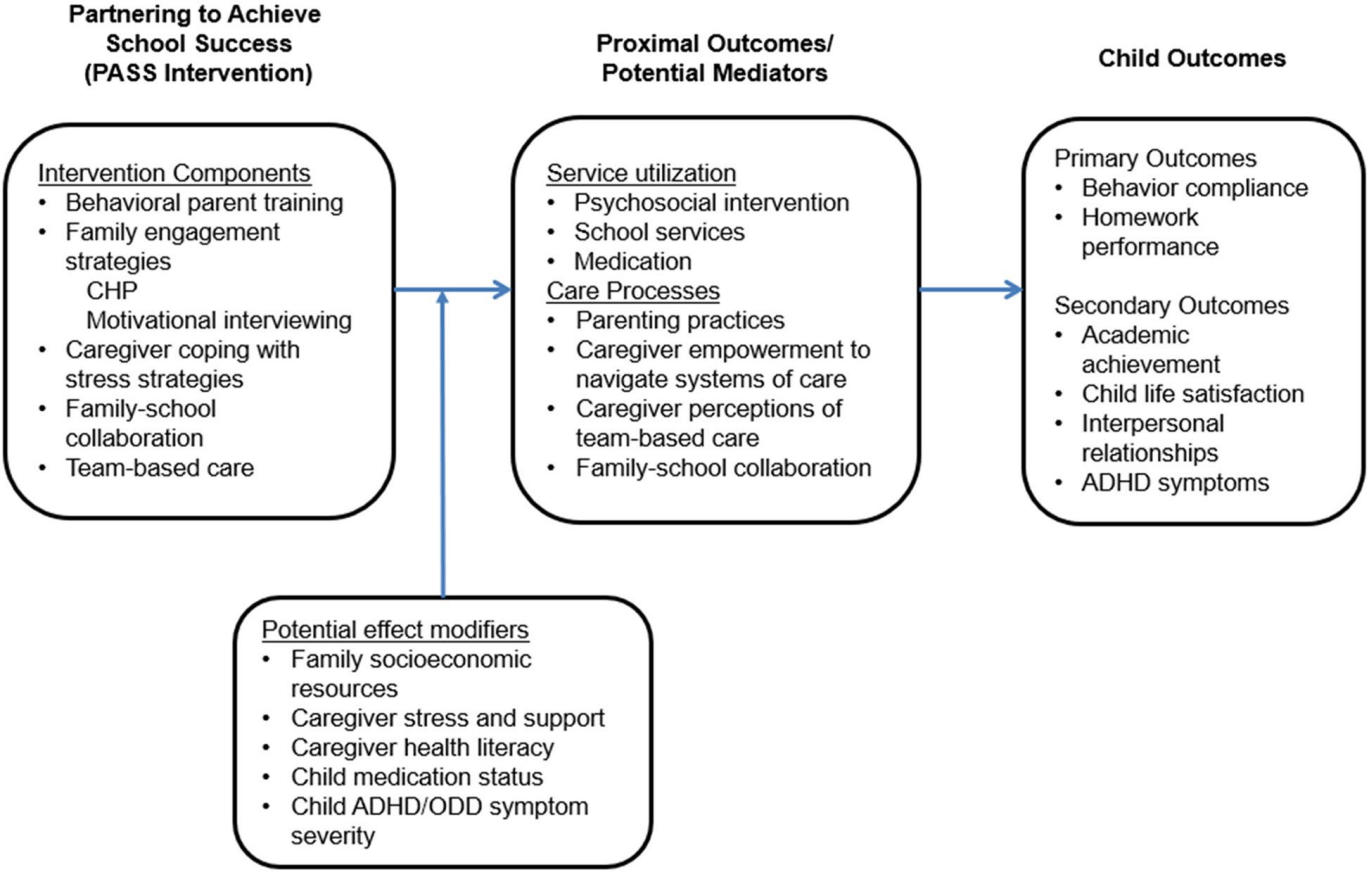
Conceptual Model for PASS intervention

The conceptual framework (Fig. 1) for the treatment comparison (PASS vs. TAU) is based upon evidence that integrating enhanced behavior therapy services into primary care improves patient-centered outcomes and increases service utilization [27]. The PASS intervention includes evidence-based behavior therapy (specifically behavioral parent training) and enhancements to promote family engagement in the intervention, caregiver ability to cope with chronic stress related to parenting and life circumstances (distress tolerance), caregiver motivation for change, family-school collaboration, and family involvement in team-based health care. Consistent with a developmental-ecological framework [31], PASS is designed to align systems of care (family, school, primary care) to promote healthy child development. PASS is based on a team-based medical and behavioral health service model that includes regular collaboration between the PASS clinician and the primary care provider (PCP). To assist families in overcoming barriers to care, sustaining their engagement in PASS, and obtaining needed community resources, PASS includes support from a Community Health Partner (described below).

According to self-determination theory [32], engagement and motivation for change are fostered by supporting caregivers’ autonomy, competence, and relatedness. PASS includes strategies to encourage caregivers’ autonomy, competence, and relatedness so that they are empowered to effectively navigate systems of care. In addition, PASS clinicians support caregiver autonomy by encouraging active involvement in team-based care that is tailored to family goals and preferences for treatment. Further, to promote caregivers’ sense of competence, PASS clinicians support consistent implementation of recommended evidence-based strategies (e.g., effective parenting practices), and partner with families to address barriers to implementation.

We propose that several factors, including family socioeconomic resources, caregiver stress and support, caregiver health literacy, and child medication status, will contribute to changes in service utilization and critical care processes (parenting effectiveness, caregiver empowerment, caregiver perceptions of team-based care, and level of family-school collaboration). In turn, we propose that changes in these proximal variables will lead to improved patient-centered outcomes (behavior compliance, homework performance, academic achievement, child life satisfaction, interpersonal relationships, and ADHD symptoms) [26, 33].

## Method

### Participants, interventions, and outcomes

#### Study setting

The study will be conducted in seven practices in the primary care network affiliated with a large children’s hospital in the Northeastern United States. These practices were chosen because they serve a relatively high percentage of economically marginalized families who disproportionately belong to underrepresented racial and ethnic groups (mostly Black/African American). See Table 1 for demographic information about each of these practices.

**Table 1:** Demographic characteristics of the targeted primary care practices

Study Site	% Medicaid	% Black/African American	% White	% Hispanic
Site A	93%	93%	2%	4%
Site B	92%	89%	4%	6%
Site C	89%	58%	26%	11%
Site D	39%	19%	69%	7%
Site E	60%	29%	61%	4%
Site F	36%	46%	35%	14%
Site G	60%	32%	36%	23%

#### Eligibility criteria

Participants will include children 5 to 11 years of age who are diagnosed with ADHD, and their parents/caregivers. The following are the inclusion criteria:

1. Child has an existing diagnosis of ADHD, as indicated in the electronic health record (EHR), and exhibits evidence of current impairment, as indicated by the parent.
2. Child is 5 to 11 years of age at time of referral.
3. Child has at least one parent or legal guardian who speaks English or Spanish.
4. Child appears to live in an economically marginalized family, as indicated by eligibility for Medicaid or Children’s Health Insurance Program in Pennsylvania or child living in a census tract or census block with median income at or below two times the federal poverty level (child’s home address extracted from EHR).

The following are the exclusion criteria:

1. Child has autism spectrum disorder and/or an intellectual disability, as reported by the referring clinician or parent, and/or indicated in the EHR.
2. Child has a comorbid condition (e.g., depression, traumatic stress) that is a major clinical concern and requires an alternative form of treatment, as reported by the parent during an initial phone interview. As a rule, children with comorbid conditions will be included.
3. Child is receiving psychological therapy from another provider at the time of recruitment.
4. Child has a sibling currently involved in the intervention or follow-up period of the study at the same time.
5. Child has received services from the integrated behavioral health team within the last 6 months (these children are considered “actively involved in care” and will be referred back to the provider of record).

#### Intervention conditions

The comparators in this study are enhanced behavior therapy (PASS) and TAU informed by AAP guidelines for ADHD [19] and facilitated by electronic practice supports, including access to integrated behavioral health services offered in the practice.

#### Partnering to Achieve School Success (PASS)

PASS is a personalized, enhanced behavioral intervention for ADHD that includes evidence-based behavior therapy strategies and enhancements to promote family engagement in treatment, increase caregiver distress tolerance [34], and improve team-based care [35]. PASS was designed based on feedback from families, behavioral health clinicians, caregivers, and educators for use with families of children with ADHD in the context of primary care practices serving primarily economically marginalized families. PASS is designed to modify contexts of development (family and school) and align systems of care (family, school, primary care) to prevent unhealthy patterns of behavior. This intervention includes regular collaboration between the PASS clinician and PCP.

PASS therapists utilize *engagement and motivation* strategies during each session to reinforce help-seeking behavior, support family empowerment, and encourage family adherence to recommended strategies. *Team-based care* has been incorporated based on feedback from our community partners and focuses on: (a) regular communication between the PASS provider and PCP and (b) the development of a problem-solving partnership between caregivers and teachers to address school problems. PASS content is delivered in a modular format based on family goals (e.g., focus on school performance vs. home behavior vs. peer relationships) as delineated in the family-centered treatment plan developed in partnership with the family and PCP. (See additional file 1 for an outline of PASS with details about core principles and intervention modules.) In addition, PASS includes the involvement of a Community Health Partner [35], a bachelor’s level staff member who contacts families by phone on an ongoing basis to: (a) promote attendance and implementation of PASS strategies, (b) assist in resolving barriers to treatment, and (c) guide families to community resources as needed. Community health partners have been shown repeatedly to be helpful in promoting engagement in services and improving the cultural effectiveness of care [30, 36].

#### Treatment as usual (TAU)

The control condition (TAU) includes access to existing integrated behavioral health service in primary care. Services provided to families in primary care during the 16-week intervention period are highly variable. The lag time from referral to first appointment and the availability of follow-up appointments after first visit vary markedly across practices. TAU does not include support from a Community Health Partner. Both conditions will include ongoing pediatric health care informed by AAP guidelines for managing ADHD [19] and facilitated by electronic practice supports, which have been successfully incorporated into the EHR to guide PCPs in implementing ADHD guidelines. Families assigned to TAU may be referred for community-based behavioral health services during the study period.

#### Training of PASS clinicians and the community health partner

The PASS clinicians generally are licensed behavioral health professionals embedded in the practices. Advanced trainees (e.g., post-doctoral fellows in psychology) receiving in-session supervision by an attending provider or additional licensed professionals are enlisted as needed to ensure enrolled families assigned to the PASS condition receive the intervention in a timely way. Each clinician will receive 4–6 h of training from a trained, experienced PASS clinician, prior to intervention. PASS clinicians will be trained to implement the treatment manual and attend to process variables, including establishing trust, listening actively, and re-directing tangential comments. In addition, a senior PASS clinician will provide 4 h of training and bi-weekly consultation with the Community Health Partner. Community Health Partner training will include an overview of the PASS intervention as well as training specific to the role of the Community Health Partner.

#### Assessment of intervention fidelity

PASS sessions will be audio recorded to enable our team to examine intervention content fidelity. The PASS treatment manual includes two standard sessions (sessions one and two) that are always presented and a variable number of additional sessions (sessions 3 +) that can be selected depending on presenting concern and family need or preference. Checklists reflecting session components will be used to assess content fidelity. Up to four sessions will be selected for content coding for each participant in the PASS condition. Sessions one and two will always be coded. One randomly selected session from sessions 3–5 and another from sessions 6 + will be selected for coding. Independent coders who have previous training in psychosocial treatment of ADHD will code all selected tapes and 25% of tapes will be double-coded to establish inter-rater reliability. An intraclass correlation coefficient (ICC) of at least 0.6 will indicate acceptable agreement [37].

The Community Health Partner will complete a survey after each encounter with a caregiver that provides information on topics discussed, barriers, stressors, strategies to address barriers and stressors, caregiver perceptions of care, resources, and the support process included in the call. To monitor Community Health Partner process fidelity, we will use a self-evaluation checklist completed by Community Health Partners after contact with a family. Process items include establishing trust, listening attentively, and re-directing tangential comments [38].

#### Measures of outcomes – examined for objective 1

Outcomes are differentiated into primary versus secondary and informant providing data (parent, teacher, child self-report). Two parent-report measures (ratings of homework performance and behavior compliance) have been identified as primary because these are a major focus of the PASS intervention. Teacher-report measures are secondary because of challenges obtaining these data due in large part to the burden placed on schools. Child self-report measures are secondary because some children, especially those under the age of 8, may have difficulty understanding and responding to these measures.

#### Primary outcomes

The Inattention/Task Avoidance factor of the *Homework Problem Checklist* (HPC) [39] will be used as a parent-report measure of child academic performance. This 12-item scale has strong psychometric properties [40] and is responsive to family-school intervention programs [41, 42]. Behavior compliance will be determined by assessing the severity of child disruptive behavior using the eight items pertaining to oppositional-defiant disorder from the *Vanderbilt Assessment Scale* parent version. The psychometric properties of parent and teacher reports on this measure have been shown to be adequate [43, 44].

#### Secondary outcomes

Behavioral health service use will be measured via a parent-report measure adapted for this study from the Service Assessment for Children and Adolescents (SACA) [45]. In this study, we will collect data on lifetime and current service utilization in outpatient mental health settings and school settings, and treatment with medication. Data gathered will include detail related to service initiation, discontinuation, and dosage.

Severity of child ADHD symptoms will be assessed using the ADHD items on the *Vanderbilt Assessment Scale* [21, 43, 44]. All nine ADHD Inattention items and three ADHD Hyperactivity/Impulsivity items will be administered. This measure will be completed by parents and teachers. Parent and teacher ratings of ADHD symptoms have been demonstrated to have excellent psychometric properties [46] and to be sensitive to change in response to treatment [47].

Academic performance will also be assessed using the *Academic Proficiency Scale* (APS) [48], which will be completed by parents and teachers. The APS assesses proficiency in academic subjects relative to standard expectations (1 = Well below standard; 3 = At standard; 5 = Well above standard). A two-subject (Math; Language Arts) version of the measure will be used in the current study.

Child relationships with peers will be examined using the *PROMIS peer relationships scales*. The child-report version consists of eight items and the parent-report measure has seven items. These measures assess the quality of children’s relationships with peers including the degree of peer acceptance. The scales have been shown to produce scores that are both reliable and valid based on analyses using item response theory [49].

Child life satisfaction will be assessed using the *PROMIS life satisfaction scale.* The child-report and parent report measures each have four items. The scale assesses children’s and parents’ evaluations of the quality of the child’s life. Using analyses based in item response theory, these scales have been shown to be reliable with a wide range of life satisfaction levels (from 2.5 SDs below the mean to 1 SD above the mean) [50].

### Measures of care processes – potential mediators – examined for objectives 2 and 3

#### Parenting practices

The *Parent–Child Relationship Questionnaire* (PCRQ) [51] will assess parent perceptions of their parenting practices. The Negative/Ineffective Discipline factor and four items from the Positive Involvement factor will be administered. This measure has been shown to be internally consistent and sensitive to the effects of behavioral intervention [41, 52].

#### Family empowerment

Parent empowerment regarding their knowledge of how to obtain services for their children will be assessed using the Service System factor of the *Family Empowerment Scale*. This twelve-item scale has been shown to have adequate reliability (alpha = 0.87; test–retest = 0.77) and validity [53].

#### Team-based care

To evaluate parental perceptions of team-based care, a seven-item scale was derived from the Clinician and Group Survey, Version 3.0 of the *Consumer Assessment of Healthcare Providers and Systems* (CAHPS, https://cahps.ahrq.gov/surveys-guidance/cg/about/index.html). The scale includes four Communication and four Care Coordination items. The psychometric properties of this scale will be examined during the study.

#### Parent-teacher involvement

The Quality of Parent-Teacher Relationship subscale of the *Parent-Teacher Involvement Questionnaire* (PTIQ) [54] will be used to evaluate the quality of the family-school relationship. The parent version of the form includes 6 items, and the teacher version consists of 5 items. Subscales have been validated in families of youth with ADHD and have demonstrated acceptable internal consistency and validity [55].

### Measures of variables for subgroup analyses (Assessed at baseline; examined for objective 4)

#### Sociodemographic factors

Parents will be asked to classify their and their *child’s race and ethnicity* and report the *primary caregiver’s highest educational attainment* at the current time. To assess *household income and poverty level*, items will be derived from the National Health Interview Survey (NHIS) Family Questionnaire (2007) that assess household income of all family members from all sources before taxes in the prior calendar year and whether the family is above or below the poverty level.

To measure *financial resource strain*, a single item from the Study of Women’s Health Across the Nation (SWAN) study will be administered to assess family ability to pay for basics such as food and housing. Prior research supports the item’s validity [56].

To assess *caregiver perceived stress,* caregivers will complete the *Perceived Stress Scale* (PSS), a 10-item measure of the degree to which respondents feel that their lives are unpredictable and overloaded in the past month. The PSS has adequate reliability and expected associations with life stressors, depression, anxiety, and somatic symptoms [57].

To examine the health literacy of caregivers, the *Short Assessment of Health Literacy* will be used [58]. This 18-item measure was developed and validated using item response theory. The internal consistency of the scale is in the acceptable range and relatively high for caregivers with lower levels of health literacy. Correlations with other measures of health literacy are relatively high, supporting the validity of the measure.

#### Medication status

We will track medication status by: (a) asking parents to report on medication status at each data collection period using the service use form, and (b) obtaining data from the EHR related to medication prescriptions and reconciliation of active medication orders. Medication status for purposes of randomization and analysis of subgroups will be determined by parent report of medication use at baseline. In addition, we will examine whether changes in medication status (based on parent report at each data collection period) during the intervention and follow-up periods (e.g., change from off to on medication) has an effect on treatment outcomes. We will use medication data obtained from the EHR to assess for gaps in medication use for patients who are on medication at the start and end of the study period.

#### Clinical characteristics

Our team will explore whether clinical characteristics of the child at baseline, such as severity of ADHD, oppositional defiant disorder (ODD), and anxiety/depression symptoms assessed using the Vanderbilt scales, have a moderating influence on the effect of intervention on child outcomes.

### Assessment of family and provider engagement in intervention

Family attendance at sessions is documented in the electronic health record. The Community Health Partner will maintain records of all contacts (phone, email, text) initiated and successfully completed with families. Parent satisfaction with PASS will be assessed using a modified version of the Treatment Evaluation Inventory [59]. Primary care provider satisfaction with PASS will be evaluated using a measure of satisfaction developed in collaboration with clinician partners and advisors. Parent/legal guardian/caregiver satisfaction with the modality of PASS delivery (i.e., in-person or telehealth) will be assessed using a novel Acceptability of PASS Delivery Modality measure adapted for the current study from the Acceptability of Intervention Measure. The Acceptability of Intervention Measure has demonstrated strong psychometric properties and is designed to be adaptable for a wide range of populations and interventions [60].

### Participant timeline

Study involvement for participants will begin at the time of family consent. The study will end for participants at the time of follow-up data collection. See Table 2 for a study timeline.

**Table 2.**
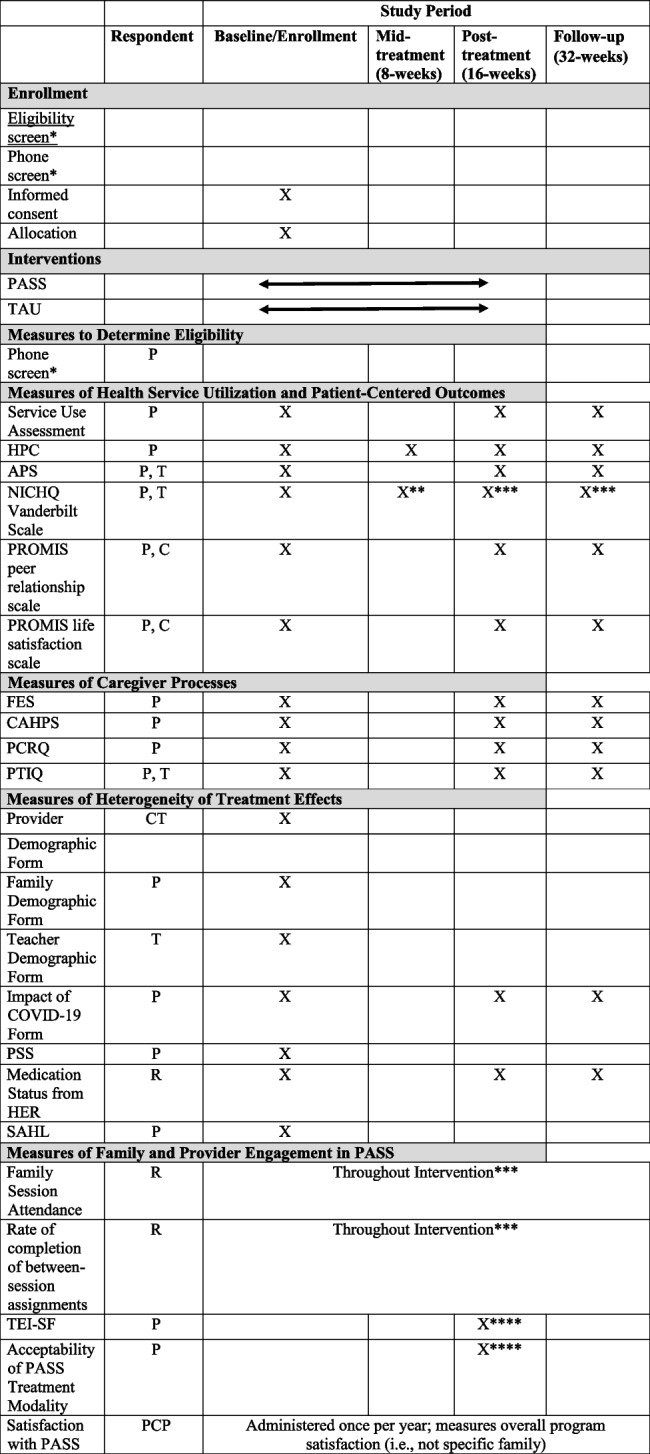
Timeline and Measure Completion

*PASS *Partnering to Achieve School Success, *TAU *Treatment as usual, *HPC *Homework Problem Checklist, Inattention/Task Avoidance subscale, *APS *Academic Proficiency Scale, *NICHQ *National Institute for Children’s Healthcare Quality, *PROMIS *Patient-Reported Outcomes Measurement Information System, *FES *Family Empowerment Scale, *CAHPS *Consumer Assessment of Healthcare Providers and Systems, Communication and Care Coordination subscales, *PCRQ *Parent-Child Relationship Questionnaire, *PTIQ *Parent-Teacher Involvement Questionnaire, *PSS *Perceived Stress Scale, *EHR *Electronic health record, *SAHL *Short Assessment of Health Literacy, *TEI-SF *Treatment Evaluation Inventory, Short Form, *P *Parent, *C *Child, *T *Teacher, *PCP *Primary Care Provider, *CT *Clinical team, *R *Research Team.

^*^Administered at screening visit prior to enrollment.

^**^Only parent-rated Oppositional Defiant Disorder Symptoms will be administered at mid-treatment

^***^Only ODD and ADHD Symptoms will be administered at post-treatment & follow-up (both parent and teacher)

^****^Measure is only administered for PASS condition

### Sample size

Based on prior research [26, 28, 35], the magnitude of the effect size reflecting the degree of change in response to PASS vs TAU for service use and child outcomes (Objective 1) and care processes (Objective 2) is expected to be the 0.35 to 0.60 SD range. Based on prior research using a similar behavioral intervention [41], we assumed a standard deviation for a single observation = 0.50 and an autocorrelation = 0.3. Projecting an enrollment of 300 participants (150 per condition), and given an expected 20% attrition rate, we expect 240 participants will provide parent-report and child self-report data at baseline and at least one other data collection point. Using statistical methods described below, analytic models will achieve 85% power to detect an effect size as small as 0.30 SD. Given the challenges of collecting data from teachers related to the COVID-19 pandemic, the number of evaluable cases will be well below 240 (estimated to be 100). As such, analyses of teacher-report data will be exploratory. In addition, analyses of mediation (Objective 3) and subgroup effects (Objective 4) will be exploratory.

### Recruitment

Participants will be referred to the study via several pathways that have been developed in collaboration with community partners, including PCPs, PASS clinicians, and parents. Clinicians in primary care, including PCPs and behavioral health clinicians, can refer potentially eligible patients to our team through the EHR. The referring clinician will inform the family about the study and, with parent permission, indicate in the EHR that the family has agreed to be contacted by the study team. Another pathway is through automated review and reporting from the EHR. Specifically, children who receive care in one of the participating practices may be identified as potential participants if their EHR indicates that they meet preliminary inclusion criteria (i.e., documented ADHD diagnosis, between 5 and 11 years old, evidence of low-income status in the EHR). Reports will be generated regularly and provided to the study team. Then the study team will reach out to the associated PCP and inquire about the suitability of the family for the study. As an additional recruitment tool, the study team has created recruitment letters and cards with a QR code that enables families to express interest in the study directly with study team members. If the family is interested, the family will complete an online questionnaire that will capture contact information. Completion of the survey will act as confirmation of willingness to be contacted by the study team to gauge interest and complete a phone screen.

Regardless of referral pathway, the study team will contact families by telephone to screen them for the study and, if potentially eligible, obtain their verbal consent to advance through the recruitment process. The screening will include requesting parents to respond to questions to verify that inclusion criteria are met and determine whether exclusion criteria are present. If families are eligible and provide verbal consent, they will be scheduled for a study visit to obtain written consent and collect baseline data. During the initial phone contact, families will be given the option to participate in the study or decline participation. If families decide not to participate, they can be referred either to the integrated behavioral health provider within their primary care practice, including brief behavior therapy with limited school collaboration and no support from a Community Health Partner, or to a community-based behavioral health provider.

**Fig. 2:**
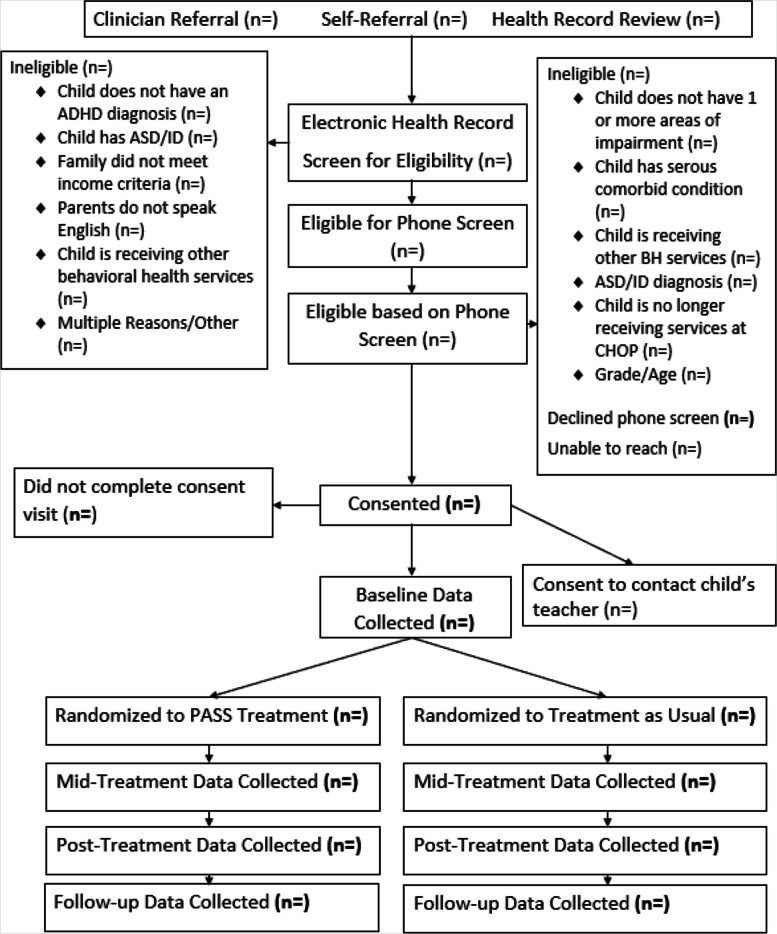
PASS Study Participant Flow CONSORT

If families participate in the study, they will be randomly assigned to PASS or TAU. With family consent, a member of the research team will contact the child’s teacher. We will use procedures successfully employed in previous studies [41] to obtain teacher buy-in: (a) an email will be sent to inform the principal that a parent or guardian has given permission for data collection from a student’s teacher, and the principal will be encouraged to contact the team if there are any concerns, (b) a member of the team will contact the teacher via phone and email, (c) teachers will be provided a “Teacher Information Sheet” that includes details of the study and a copy of the signed consent form giving parent permission to obtain data from the teacher, and (d) the study team will ask the teacher to complete baseline measures and a brief demographic form. Similar procedures will be used for data collection at other assessment points. See Fig. 2 for an illustration of participant flow through the study.

### Assignment of interventions

Participants will be randomly assigned to condition at a 1:1 ratio. Randomization will be stratified by child medication status (on or off ADHD medication at the time of consent) and practice site (seven practices).

#### Sequence generation

The sequence of assignments to treatment conditions will be generated using a computerized randomization algorithm, ensuring an unbiased and unpredictable assignment of participants to the study arms. The randomization list will be securely uploaded into REDCap (Research Electronic Data Capture) [61, 62].

#### Allocation concealment mechanism

Randomization will occur after informed consent is obtained from parents/legal guardians and assent is obtained from children (if indicated). We will use the randomization module in REDCap, so there will be no opportunity for study team members to become aware of group allocation until the computer reveals the result. A study statistician will generate the allocation table using statistical software and upload the table into REDCap without sharing the table with any research team members.

#### Implementation

Randomization procedures including allocation sequence will be generated by a study statistician. For each participant, the allocation will be revealed at the time of enrollment. A study team member will inform parents/legal guardians and children about treatment assignment after obtaining consent and assent (if indicated).

#### Concealment

Given that the study is a pragmatic trial being conducted under real-world conditions, no attempt will be made to conceal treatment assignment condition from parents/legal guardians and children. In addition, no attempt will be made to conceal treatment assignment from study team members, PCPs, behavioral health clinicians, and teachers.

#### Data collection and management

Data collection strategies were designed in partnership with caregiver, educator, and clinician partners. The strategies will be reviewed and modified throughout the study to maximize participant engagement in data collection and minimize missing data. Baseline data will be collected from parents and children at the time of consent, and baseline data will be collected from teachers shortly thereafter. Mid-treatment data (specifically the ODD items from the Vanderbilt scale and the Inattention/Task Avoidance items of the Homework Problem Checklist) will be collected from parents approximately 8 weeks after baseline, and post-treatment data will be collected from parents and teachers 16 weeks after baseline. Follow-up data will be collected from parents and teachers 32 weeks after baseline. Study data will be collected and managed using REDCap tools hosted at our institution [61, 62].

#### Plans to address missing data

Numerous strategies will be applied to minimize missing data, including persistent efforts to contact parents and teachers, disseminating study newsletters on a periodic basis, and motivational interviewing strategies. Alternative methods of imputation, including the maximum likelihood method [63] and multiple imputation method will be considered. In addition, we will check whether patterns of missingness differ between the PASS and TAU groups to determine whether the amount of missingness is associated with a particular group.

## A priori plans for data analysis for each objective

### Objective 1 (Evaluate differences between PASS and TAU in improving service utilization and child health outcomes)

To evaluate changes in service utilization, we will examine between-group changes in the proportion of children receiving mental health services (i.e., outpatient mental health therapy, school mental health services and special education services, and psychotropic medication) from pre-treatment to post-treatment to follow-up using the McNemar test. To evaluate the effect of intervention on child outcomes, a mixed effects model with repeated measures (MMRM) will be the general analytical approach employed. The model will examine intervention effects across four time points (baseline, mid-treatment [parent report of homework performance and behavior compliance only], post-treatment, follow-up). Data for all participants randomized to treatment condition will be included in the analytic model, consistent with an intent-to-treat (ITT) approach. Mixed effects models will estimate within-subject effects by time and between-treatment effects, as well as the interaction of time by treatment. A significant time by treatment interaction will indicate that one treatment is superior to the other. MMRM will include the fixed categorical effects of treatment, time, randomization stratification factor (medication status and primary care practice), and treatment-by-time interaction as well as covariates as indicated. Within-participant error variance–covariance will be assumed to be an unstructured matrix. Point estimates and confidence intervals for the mean change in outcomes for each treatment group as well as the difference in the estimated mean change between groups will be calculated.

Most of the outcome domains examined under Objective 1 include two measures. For example, there are two primary outcome measures – parent report of homework problems and parent report of behavior problems. In addition, ADHD symptoms will be assessed by parent report of inattention and hyperactivity-impulsivity symptoms. Because each domain generally will be assessed using two measures, tests of significance within each outcome domain will be examined using an adjusted *p* value. We have elected to use the conservative Bonferroni approach, and therefore will use a p value of 0.05/2 = 0.025. As indicated, analyses of measures completed by teachers will be underpowered. We will conduct these analyses using a* p* value of 0.05, but the primary focus will be on evaluating the magnitude of the effect size.

### Objective 2 (Evaluate differences between PASS and TAU in changing parenting practices, improving family empowerment in seeking services, and improving caregiver perceptions of team-based care)

MMRM using an ITT approach will be the general analytical approach for testing Objective 2. The analytic model for examining Objective 2 will examine intervention effects across three time points (baseline, post-treatment, follow-up). We will test for significance using a *p* value of 0.025 to be consistent across analyses.

#### Objective 3 (Explore whether changes in parenting practices, family empowerment, and perceptions of team-based care mediate the effect of intervention on child outcomes)

We will examine whether changes in parenting practices, family empowerment, or perceptions of team-based care mediate the effect of treatment (PASS, TAU) on child outcomes. We will employ the method proposed by Hayes for examining mediation effects with regression modeling approaches, utilizing PROCESS macro [64]. The models will reflect change scores in the mediators. To produce a precise estimate of mediation effects and properly account for the potential of regression to the mean, baseline measurement of the mediator will be included as a covariate [64]. In each analysis, we assess mediation by first estimating the effect of the intervention condition (PASS, TAU) on the child outcome at post-treatment, while controlling for the influence of the mediator and estimating the direct effect of the intervention on child outcomes without the mediator. The mediation effect is the difference in estimated intervention effects between the models. If the difference is not equal to zero, then there is evidence of mediation of the intervention on child outcomes.

#### Objective 4 (Examining Heterogeneity of Treatment Effects; HTE)

Our general strategy is to determine whether the effect of treatment on outcomes varies as a function of baseline level of family resources, caregiver health literacy and stress, child medication status, and child mental health symptom severity at baseline, following the recommendations of Wang and Ware [65]. We will use a regression modeling approach with the outcome being change from baseline to post-treatment/follow-up, treatment groups being the independent variable, a subgrouping variable or covariate being the moderator tested, and the test of moderation being the interaction of treatment by the covariate. A significant interaction term indicates that treatment varies as a function of subgroup. For a subgroup variable with more than two levels (e.g., caregiver stress), the presence of a significant treatment by subgroup variable interaction will be followed by a series of post hoc analyses to conduct pairwise group comparisons. The *p* values for post hoc analyses will be used to calculate Bonferroni adjusted *p* values and false discovery rates (FDR) resulting from multiple comparisons [66, 67]. Given that HTE analyses will likely be underpowered, a consideration of Bonferroni adjusted values and FDR in the discussion of the heterogeneity of treatment effects will be informative.

## Plan for Sensitivity Analyses to Determine Impact of Key Assumptions

### Objective 1 and objective 2

As a sensitivity analysis, we will use a Generalized Estimating Equation (GEE) approach to analyze Objectives 1 and 2. Like MMRM, GEE is based on regression techniques and uses all data obtained from each participant. Potential advantages of GEE are that it is suitable for both continuous and dichotomous outcomes, and it does not require the correct specification of the variance–covariance structure. Additionally, GEE can be used to model data with nested structures by accounting for the correlation among outcomes for individuals in the same aggregate.

#### Objective 3

We will explore a multiple mediator model using structural equation modelling. The multiple mediator model posits a path model for each additional mediator and estimates a separate effect for each mediator. The significance of the mediation effects can be tested using a Sobel test based on an asymptotic approximation to the distribution of the indirect mediation effects [68, 69]. However, because this test is sensitive to sample size, as an alternative sensitivity analysis for mediation analyses, we also plan to use a non-parametric bootstrapping approach. Additional methods based on simulations will also be considered [70, 71].

#### Objective 4

A simultaneous assessment of mediator–moderator effects will be examined. We will examine mediated-moderation effects and moderated-mediation effects using strategies outlined by Fairchild and MacKinnon [72] and Hayes [73]. These strategies extend the mediator and multiple mediator models described under Objective 3 by adding a moderator effect and an interaction between treatment by moderator effect to the main models. Tests of significance for the individual moderator, mediator and interaction effects can be conducted using tests of joint significance and similar bootstrapping methods as for mediation effects.

#### Determination of intervention effect sizes

To estimate the magnitude of the intervention effect, we will compute Cohen’s *d*. This statistic will be calculated as the ratio of between-group differences in changes in expected values over time divided by the standard deviation of the combined, unadjusted baseline scores for each outcome measure.

#### Data monitoring

The project statisticians will monitor data security on an ongoing basis. Oversight for data security and participant safety will be provided by a four-member Data and Safety Monitoring Board (DSMB). This board includes three senior-level faculty members, one of whom is a professor of biostatistics, and another who is a leader of our institution’s Research Family Partners Program and serves as a family advocate. This group will convene twice yearly. Included in its responsibilities, the DSMB can request an interim analysis to determine whether it is justifiable to continue the clinical trial, if questions about the rights and safety of participants arise.

#### Harms

The study team will inform the IRB of serious adverse events in a timely manner. Other adverse events will be documented in study records as directed by the IRB.

#### Auditing

The Office of Research Compliance at our institution audits study processes and procedures on an ongoing basis and is available to the study team for consultation as needed.

#### Protocol amendments

Amendments to the study protocol will be submitted to the IRB for review and approval.

#### Confidentiality

All data generated during this study will be kept confidential in accordance with institutional policies and HIPAA. Participants will be identified in the study database by an ID number. Electronic files holding the link between study ID number and identifying information will be kept in password-protected files kept on the secure study drive. The files linking study IDs and private health information will be retained for a minimum of 6 years post the data collection phase in accordance with our institution’s data retention policy.

#### Ancillary and post-trial care

Families who still need care after the study period will be able to continue with the integrated behavioral health provider in their primary care practice, or they will be referred for community-based services. If interim care is needed to address urgent issues, study clinicians will be available to provide support.

#### Dissemination policy

We will disseminate research methods and findings to researchers and practitioners in pediatrics, school and clinical psychology, and school mental health through presentations and publications in peer-reviewed journals. A summary of study results will be posted to the study website: https://www.research.chop.edu/partnering-to-achieve-school-success-study. Further, in collaboration with our institution’s Communications and Public Relations teams, we will publicize the results to families, teachers, and intervention providers through press releases and notifications on our institution’s Facebook pages and Twitter and LinkedIn feeds.

## Discussion

The major goal of this study is to evaluate the effectiveness of enhanced behavior therapy implemented in pediatric primary care tailored for families of children with ADHD who are economically marginalized. Enhancements include strategies to promote family engagement by involving Community Health Partners, a focus on distress tolerance to support caregivers in coping with chronic stress, team-based care involving collaboration between health and mental health clinicians, and strategies to promote effective family-school collaboration.

Strengths of the study include: (a) the intentional targeting of children and families who historically have been underrepresented in intervention trials; (b) examination of an intervention that has been uniquely tailored to address the needs of children and families marginalized by poverty who disproportionately belong to underrepresented racial/ethnic groups; (c) implementation of a clinical trial under conditions similar to authentic community-based practice; and (d) examination of outcomes important to the families of children with ADHD. In addition, this study will explore factors, such as parenting practices, family sense of empowerment to seek and obtain needed services, and caregiver perceptions of team-based primary care, that might explain in part the effect of intervention on child outcomes. Further, this study will examine whether there are differential effects of intervention based on socioeconomic factors and clinical characteristics of the child.

Outcomes of this study will be assessed using informant-report measures completed by parents, teachers, and children themselves. A practical challenge is that it can be difficult to obtain data from teachers. In some cases, caregivers might not provide consent for study team members to collect data from school personnel. In other cases, it might be difficult to connect with teachers and obtain their consent. Even when teachers are willing to participate, they might not complete measures, likely because of limited time and competing priorities when working in schools with limited resources. Nonetheless, we anticipate that we will have some success in collecting outcome data from teachers, which will enable our team to explore intervention effects on teacher-reported outcome measures.

Another practical challenge is that most of the behavioral health clinicians who will provide PASS work in large primary care practices with a high volume of referrals for behavioral health services. Although these clinicians often can provide brief same-day behavioral health care when children are seen for medical appointments, it can be difficult for them to offer follow-up care in a timely manner. By consenting to participate in this study, these behavioral health clinicians will agree to integrate PASS study cases into their busy schedules. By so doing, there might be constraints on how many appointments they can schedule with PASS patients during the 16-week intervention period.

The findings of this study will inform clinicians and researchers about how to provide psychosocial intervention in primary care to children with ADHD and their families who are economically marginalized. In addition, the study will identify strategies for overcoming barriers to providing evidence-based behavioral health services in pediatric primary care, informing more practical and sustainable models of care.

### Supplementary Information

Supplementary Material 1.

## Data Availability

The datasets and materials used during the current study are available from the corresponding author on reasonable request.
