# Supplementary material for: Reducing disparities in behavioral health treatment in pediatric primary care: a randomized controlled trial comparing Partnering to Achieve School Success (PASS) to usual ADHD care for children ages 5 to 11 – study protocol
Source: BMC Prim Care. 2024 Jun 22;25:225. doi: 10.1186/s12875-024-02473-7 (PMC11193903; doi:10.1186/s12875-024-02473-7)
Supplement: Supplementary file 1 — Supplementary Material 1. [file 12875_2024_2473_MOESM1_ESM.docx]

**Partnering to Achieve School Success (PASS) Intervention Outline**

**PASS Core Principles:**

1. A strong parent-child relationship is critical for healthy child development.
2. Strong teacher-student relationships are critical for success in school.
3. Promoting health habits increases children’s ability to perform well in school and lead a healthy life.

**Enhanced Family Involvement Strategies (Engagement and Motivation Strategies)**

The PASS clinician attempts to address these goals/integrate these strategies into all PASS sessions. In addition, the Community Health Partner is involved in supporting family involvement in PASS, as indicated below.

Goals:

1. Reinforce help-seeking behavior
   1. Understand family’s help-seeking history
   2. Identify past facilitators and barriers
2. Utilize motivational interviewing strategies to encourage family engagement in care and motivation to change/address challenges
   1. Four General Principles of Motivational Interviewing (Miller & Rollnick, 2023)
      1. Express empathy
         1. Foster a family-centered style of therapy.
         2. Communicating acceptance will facilitate the process of change.
         3. Use reflective listening to help understand how the parent feels and views concerns/issues without judging, criticizing, blaming. Express acceptance for the patient “as they are.”
         4. Ambivalence about changing should be expected and accepted
      2. Develop discrepancy
         1. The arguments and rationale for change must come from the parent
         2. Help identify a discrepancy between a parent’s goals/values and current behavior
         3. Establishing the discrepancy is related to the importance of the change desired – highlighting the discrepancy will help overcome ambivalence by sparking intrinsic motivation (rather than change that relies on someone else’s goals)
      3. Roll with resistance
         1. Disengage with arguments against change.
         2. Do not oppose resistance.
         3. New ideas can be accepted but not imposed by the therapist. The parent is the primary resource for determining solutions to problems
         4. Resistance is a signal to the therapist to respond differently.
      4. Support self-efficacy
         1. The parent’s belief in the capacity for change (hope) is a critical source of motivation.
         2. The therapist’s belief in the parent’s ability to change may be a self-fulfilling prophecy
         3. The message should be: “If you wish, I can help you change.”
3. Affirm family engagement and efforts to implement recommended strategies/support the child
4. Acknowledge that parental stress impacts how adults care for their children. Provide opportunities to discuss stressors, offer support, and discuss strategies for caregiver distress tolerance.

Community Health Partner Tasks:

1. Contact the family by phone about twice per month during the intervention period and after each missed session.
2. Remind the family about upcoming sessions and discuss potential barriers to attendance.
3. Encourage the family to complete between-session homework assignments. Discuss barriers to implementation. Share information about implementation challenges with PASS therapist (document telephone encounter in the EHR).
4. Communicate with families about useful community resources (e.g., after school tutoring programs).
5. Respond to family questions about community resources. Interface with practice-based social workers as needed (e.g., to address housing or other services questions).

**Team-based Care Strategies (Integrated Medical and Behavioral Health Care)**

Goals:

1. Establish a collaborative relationship with the PCP and review common factors
   1. Notify PCP that family has consented to treatment; provide overview of PASS
   2. Encourage PCPs to listen with empathy, instill hope, and obtain permission from families to discuss sensitive issues
2. Establish a family-centered treatment plan
   1. After the initial PASS session with the family, share preliminary treatment plan with the PCP (route initial PASS session note to PCP)
   2. Encourage the PCP to identify additional goals in preparation for conjoint session with family
   3. Remind PCP about team-based care expectations and communication plan (e.g., communications through EHR, expected timeline, method to contact therapist if needed).
3. Provide feedback to PCP throughout the intervention about changes in treatment plan or if the child/family experiences a significant setback.
   1. If the PCP is managing medication and there is a medication concern, notify PCP through EHR. Collaborate with PCP to monitor child response to medication.
   2. If there are concerns about family attendance/adherence:
      1. Notify PCP about concerns (use EHR and discuss in person)
      2. Collaborate with PCP to develop a strategy to encourage attendance/adherence
      3. When warranted, involve the practice-based social worker (at PCP’s direction)
4. At the conclusion of treatment, complete discharge/transfer summary document and route to PCP in EHR.

**PASS Intervention Modules**

PASS intervention modules are selected and implemented based on family-identified goals for treatment and according to the family-centered treatment plan. Relevant modules are then reviewed during each PASS session (i.e., family intervention and family-school intervention content may be addressed during the same session). The following is a list of goals to be accomplished within each intervention module. These goals are addressed as relevant for each family throughout multiple sessions.

**Module 1: Evidence-based Behavioral Parent Training**

Goals:

1. Educate parents about ADHD
2. Discuss the importance of a positive parent-child relationship
3. Describe positive attending strategies
4. Present the components of The Child’s Game
5. Model and practice The Child’s Game with clinician feedback
6. Assist parents in analyzing the antecedents and consequences of behavior
7. Educate parents about effective ways of giving instructions to their child.
8. Introduce parents to basic consequence interventions for behavior management.
9. Discuss positive reinforcement and types of reinforcers
10. Describe and develop a token economy system
11. Educate parents about punishment.
12. Train parents to use punishment strategically.

**Module 2: Health Promotion/Team-based Care**

Goals:

1. Describe the importance of collaboration with the child’s medical team
2. Identify problems and resources
3. Describe ways to foster connections with primary care
4. Discuss healthy behavior and the impact on child development (e.g., healthy sleep routine)

**Module 3: Family-School Collaboration**

Goals:

1. Review the impact of ADHD on school performance
2. Prepare for teacher contact
3. Monitor the family-school relationship
4. Discuss the importance of positive student-teacher and family-school relationships
5. Review strategies for building partnerships with teachers
6. Provide psychoeducation about Section 504 Plans and Individualized Education Plans
7. Provide instruction in establishing a daily report card (DRC)

Phone Contact with Teacher:

Attempt to schedule a time for communication with the teacher early in intervention (e.g., during teacher prep periods). Obtain teacher’s email address to maintain contact and allow for follow up communication/rescheduling if phone calls are missed. The Community Health Partner may also support communication with the teacher (e.g., obtaining signed release of information from families, scheduling phone calls with teacher, obtaining teacher feedback about student performance).

Goals:

1. Describe the goals and “big ideas” of PASS – provide handouts
2. Obtain information about the child’s school performance. Discuss child strengths and needs.
3. Discuss the family-school partnership and problem solve barriers to effective collaboration
4. Review the use of the DRC when relevant
   1. Discuss implementation and adherence. Problem-solve barriers.
   2. Determine whether child is meeting goals. Consider whether goals should be adjusted and collaborate with teacher to adjust as necessary.
5. Encourage the teacher to follow up by phone if questions or concerns arise.
